# A Case Report of Acute Transverse Myelitis Following Novel Coronavirus Infection

**DOI:** 10.5811/cpcem.2020.5.47937

**Published:** 2020-05-12

**Authors:** Deesha Sarma, Leslie A. Bilello

**Affiliations:** Harvard Medical School, Beth Israel Deaconess Medical Center, Department of Emergency Medicine, Boston, Massachusetts

**Keywords:** COVID-19, transverse myelitis, autoimmune, coronavirus

## Abstract

**Introduction:**

During the coronavirus disease 2019 (COVID-19) pandemic, emergency providers are not only seeing an increasing number of patients with COVID-19 infections, but also associated complications and sequelae of this viral illness.

**Case Report:**

We present the case of a 28-year-old female patient who presented after a confirmed COVID-19 infection with lower back pain, bilateral symmetric upper and lower extremity numbness, and urinary retention. The patient was diagnosed with acute transverse myelitis. She required intravenous corticosteroids and plasma exchange with significant improvement in symptoms and minimal residual effects.

**Conclusion:**

This case illustrates the importance of prompt recognition and treatment of sequelae of COVID-19 infections.

## INTRODUCTION

The novel coronavirus pandemic has resulted in significant mortality and morbidity, with almost two million cases and over 100,000 deaths worldwide as of mid-April 2020.^1^ Coronavirus disease 2019 (COVID-19), the illness caused by the severe acute respiratory syndrome coronavirus 2 (SARS-CoV-2), presents similarly to other viral respiratory illnesses with common symptoms including fever, cough, fatigue, myalgias, and diarrhea. Some patients develop respiratory distress requiring supplemental oxygen or ventilator support, while others have mild cases without complications. Given the recent and rapidly progressing nature of the pandemic, exact statistics are unknown, although estimates suggest that 80% of patients experience mild illness.^2^ Less appreciated still are the complications, sequelae, and long-term effects of COVID-19. We present a case of acute transverse myelitis following COVID-19 infection in a young and otherwise healthy patient.

## CASE REPORT

A 28-year-old female with a history of hypothyroidism on levothyroxine developed symptoms of productive cough, low-grade temperatures, low back pain, myalgias, and rhinorrhea during the novel coronavirus pandemic. She tested positive for COVID-19 via an at-home swab ordered by her primary doctor. Her upper respiratory symptoms resolved over the next week, but her lumbosacral back pain persisted and worsened, although without radiation. In addition, she developed paresthesias in her lower extremities, which progressed to total loss of sensation and with ascension of symptoms up to her mid-chest below the nipple line and bilateral upper extremities, as well as numbness to the tip of her tongue. She also reported approximately 48 hours of urinary retention as well as nausea and vomiting. She did not have any headaches, dysarthria or dysphagia, vision changes, or dyspnea. She was admitted to a large, academic, tertiary care center in Denmark.

The patient’s neurologic exam was notable for symmetrically decreased sensation below the fifth thoracic vertebra level but preserved two-point discrimination and lower-extremity motor strength. She experienced decreased proprioception and four out of five strength in bilateral upper extremities with intact reflexes throughout. Lhermitte’s sign (electric shock-like sensation down the back triggered by bending the head forward) was positive and she had a wide-based gait. She retained 1.4 liters (L) of urine in her bladder, which was relieved after Foley catheter insertion. Lumbar puncture showed 125/per microliter (/μl) mononuclear cells (laboratory reference range 0–5/μl); 0.6 grams per liter (g/L) protein (laboratory reference range 0.15 to 0.6 g/L)); normal glucose (laboratory reference range 45–80 milligram per deciliter); negative antibodies; and gram stain and culture negative for infection. Magnetic resonance imaging (MRI) with and without contrast of the cervical, thoracic, and lumbar spine showed widespread elongated signal changes throughout the spinal cord to the conus medullaris and involving the medulla, with no disc pathology or spinal canal narrowing. These findings were consistent with longitudinally extensive acute transverse myelitis (given involvement of more than three spinal cord segments), thought to be reactive in the setting of recent COVID-19 infection. The patient was started on prednisolone and received two plasma exchange treatments with rapid improvement of symptoms. After eight days in the hospital, she was discharged on a steroid taper with improved symptoms including normal urinary function. Her residual symptoms included decreased sensation in the lower extremities up to the mid-thighs bilaterally.

## DISCUSSION

Transverse myelitis is a rare, acquired neurologic condition characterized by focal inflammation and injury of the spinal cord. There is a wide array of potential etiologies. Transverse myelitis is a recognized complication of viral or bacterial infections, although it can also be the first sign of neurologic conditions such as multiple sclerosis or neuromyelitis optica or associated with systemic autoimmune diseases such as lupus or sarcoidosis.^3^ Despite extensive workup, as many as 60% of cases may remain idiopathic, meaning the exact pathophysiology of the disease is unknown and varies based on etiology.^4^ When related to an infectious cause, it is often attributed to an autoimmune-mediated response as opposed to direct invasion and injury of the spinal cord.^5^ In these cases, intravenous corticosteroids are started immediately to suppress the inflammatory response and plasma exchange is a potential treatment option to remove auto-antibodies.^6^

This patient presented with the hallmark symptoms of transverse myelitis including bilateral symmetric sensory changes and extremity weakness, lower back pain, and bladder dysfunction, and had classic contrast-enhancing lesions on MRI. Given the onset of these findings in the setting of a confirmed COVID-19 case, as well as her marked improvement with steroids and plasma exchange, it is likely that this was an autoimmune-mediated response to the novel coronavirus. Furthermore, she had no visual symptoms such as eye pain or vision loss that are classically seen in multiple sclerosis or neuromyelitis optica, nor the immunoglobulin G auto-antibodies or oligoclonal bands that are the immunological hallmarks of these diseases.^7^ Likewise her anti-nuclear antibody test, very sensitive for autoimmune disorders including lupus, was negative, nor did she have any other system involvement such as skin rash or nodules, cardiac arrhythmias, or arthritis, which are also seen with conditions like lupus or sarcoidosis.^8^

**CPC-EM Capsule**

What do we already know about this clinical entity?

Transverse myelitis is a rare neurological condition causing inflammation of the spinal cord and is believed to develop via autoimmune mechanisms.

What makes this presentation of disease reportable?

Transverse myelitis is a known sequelae of viral illnesses such as influenza, but has heretofore not been associated with novel coronavirus infections.

What is the major learning point?

Patients can develop serious complications with lasting neurological effects even after initial recovery from novel coronavirus infection.

How might this improve emergency medicine practice?

Emergency providers can prevent significant morbidity by recognizing post-infectious complications of novel coronavirus, including transverse myelitis.

## CONCLUSION

As the number of COVID-19 infections continues to rise, more patients are presenting to the emergency department with novel coronavirus-related symptoms and associated complications. Healthcare workers, especially emergency providers on the frontlines, treat these affected patients and bear witness to their different presentations and clinical courses. This case report emphasizes the importance of remaining cognizant of the atypical and less-prevalent sequelae of viral infections in patients with recent or concurrent COVID-19 infections, as prompt recognition and management are important to prevent significant morbidity. It also highlights that young and otherwise healthy patients who have seemingly recovered from COVID-19 infection can still develop serious complications. These are important takeaways for emergency providers in the midst of providing care during this global pandemic.

## References

[1] World Health Organization (2020). Coronavirus 2019 disease (COVID-19) situation report - 85.

[2] World Health Organization (2020). Coronavirus 2019 disease (COVID-19) situation report - 41.

[3] Jacob A, Weinshenker BG (2008). An approach to the diagnosis of acute transverse myelitis. Semin Neurol.

[4] Sui V, Seals M, Pope B (2019). Idiopathic transverse myelitis: observations from 60 clinical cases at UAB. Neurology.

[5] Kerr DA, Ayetey H (2002). Immunopathogenesis of acute transverse myelitis. Curr Opin Neurol.

[6] West TW (2013). Transverse myelitis - a review of the presentation, diagnosis, and initial management. Discov Med.

[7] Jarius S, Wildemann B (2013). The history of neuromyelitis optica. J Neuroinflammation.

[8] Loke WSJ, Herbert C, Thomas PS (2013). Sarcoidosis: immunopathogenesis and immunological markers. Int J Chronic Dis.

